# Microbial Community Restructuring Under Zinc Oxide Nanoparticle Stress in Membrane Bioreactors

**DOI:** 10.1111/1758-2229.70413

**Published:** 2026-09-16

**Authors:** Dongqing Zhang, Tao Yu, Antoine Prandota Trzcinski

**Affiliations:** ^1^ Nanyang Environment & Water Research Institute, School of Civil and Environmental Engineering Nanyang Technological University Singapore Singapore; ^2^ Department of Civil and Environmental Engineering University of South Florida Tampa Florida USA; ^3^ Department of Chemical Engineering Imperial College London London UK; ^4^ School of Science, Engineering and Digital Technologies, University of Southern Queensland Toowoomba Queensland Australia

**Keywords:** ecotoxicity, extracellular polymeric substances, membrane bioreactor, microbial community dynamics, soluble microbial products, zinc oxide nanoparticles

## Abstract

Zinc oxide nanoparticles (ZnO NPs) are increasingly released into aquatic environments, raising concerns regarding their impacts on biological wastewater treatment systems. This study investigated the long‐term effects of ZnO NP exposure on microbial community dynamics and extracellular responses in submerged anoxic–aerobic membrane bioreactors (MBRs) operated continuously for 144 days. Two identical MBRs were operated under control and ZnO NP exposure conditions (1–50 mg L^−1^). Reactor performance, extracellular polymeric substances (EPS), soluble microbial products (SMPs), molecular weight (MW) distribution, fluorescence characteristics and bacterial community structure were evaluated using high‐performance liquid chromatography–size exclusion chromatography (HPLC–SEC), fluorescence excitation‐emission matrix (FEEM) spectroscopy and 16S ribosomal RNA (16S rRNA) sequencing. ZnO NP exposure significantly reduced ammonium removal efficiency from 85.0% ± 2.5% to 65.2% ± 5.7% at 50 mg L^−1^, indicating inhibition of nitrification. Both LB–EPS and TB–EPS increased substantially under nanoparticle stress, while HPLC–SEC and FEEM analyses revealed accumulation of high‐molecular‐weight, protein‐like and fulvic‐like SMP fractions. Long‐term ZnO NP exposure restructured microbial community composition and was associated with reduced taxonomic and phylogenetic richness and substantial reductions in important nitrogen‐transforming genera such as *Nitrospira* and *Dechloromonas*. Overall, long‐term nanoparticle exposure promoted microbial community restructuring and enhanced extracellular stress responses, while simultaneously impairing important biological treatment functions.

## Graphical abstract

Long‐term ZnO nanoparticle exposure induced extracellular stress responses and restructured microbial communities, with reduced taxonomic and phylogenetic richness and loss of key nitrogen‐transforming taxa. These ecological changes were accompanied by impaired nitrification, demonstrating that prolonged ZnO exposure can compromise the functional stability of membrane bioreactors.

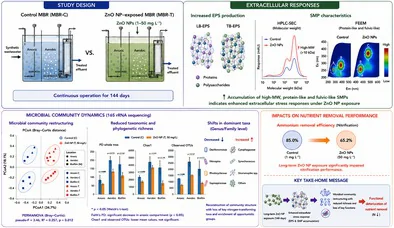

## 1 Introduction

The expanding use of engineered nanomaterials in commercial and industrial applications has increased the likelihood of nanoparticle release into natural and engineered aquatic environments, generating growing concern regarding their ecological impacts (Gottschalk et al. 2009; Limbach et al. 2008). Zinc oxide nanoparticles (ZnO NPs) are extensively produced because of their application in products such as electronics, coatings, textiles, cosmetics and packaging materials (Franklin et al. 2007). The increasing consumption of ZnO NPs has resulted in their growing occurrence in municipal and industrial wastewaters, positioning wastewater treatment plants (WWTPs) as critical interception points before environmental discharge (Ganesh et al. 2010). While ZnO NPs can be partially removed through adsorption onto sludge flocs and aggregation processes, their persistence within biological treatment systems may still disrupt microbial processes and sludge properties (Ma et al. 2014; Tan et al. 2015).

Membrane bioreactors (MBRs) are increasingly used for advanced wastewater treatment because of their high effluent quality, compact footprint and effective biomass retention. The performance of MBR systems strongly depends on the activity, stability and diversity of complex microbial communities responsible for organic matter degradation and nutrient removal. In addition to governing treatment performance, microbial communities also regulate the production of extracellular polymeric substances (EPS) and soluble microbial products (SMPs), which influence sludge properties, floc stability, microbial interactions and extracellular stress responses under environmental perturbation (Sheng et al. 2010; Jarusutthirak and Amy 2006). EPS are commonly divided into loosely bound (LB–EPS) and tightly bound fractions (TB–EPS), both of which contribute to floc stability and protection of microorganisms under stress conditions. Under adverse conditions, microorganisms may alter EPS production and composition as a defensive strategy to mitigate toxic effects and maintain cellular integrity (Sheng et al. 2010).

ZnO nanoparticles can affect microorganisms through several interconnected mechanisms, including particle–cell interactions, oxidative stress, membrane disruption and dissolution of the particles with subsequent release of Zn^2+^ ions. Importantly, ZnO is not chemically inert in aqueous environments, and its dissolution can substantially contribute to the observed toxicity. Previous studies with activated sludge have shown that soluble Zn can exert stronger inhibitory effects than particulate ZnO and that inhibition of biological processes, particularly ammonia oxidation, may be largely attributable to Zn^2+^ released during ZnO dissolution (Zheng, Wu, et al. 2011). Similarly, investigations of wastewater biofilms have demonstrated that ZnO NPs can adsorb onto biofilm surfaces while simultaneously releasing Zn^2+^, with dissolved Zn identified as an important contributor to inhibition of microbial respiration (Hou et al. 2016). More recent evidence continues to identify dissolution‐mediated Zn^2+^ exposure as a major component of ZnO NP toxicity, although particle‐associated mechanisms, including membrane interactions and reactive oxygen species (ROS) generation, may also contribute depending on environmental conditions and microbial physiology. The relative contributions of dissolved Zn^2+^ and particle‐specific effects are therefore strongly dependent on the physicochemical environment. Factors such as pH, ionic strength, organic matter, EPS and nanoparticle aggregation can alter ZnO dissolution, particle retention and Zn bioavailability. In biofilm and activated‐sludge systems, EPS may additionally bind Zn^2+^ and retain ZnO particles, thereby limiting their penetration into deeper biofilm layers while creating localised zones of elevated Zn exposure. Consequently, ZnO NP toxicity in biological wastewater treatment systems should be considered as the combined outcome of nanoparticle transformation, dissolved Zn^2+^ exposure, particle–cell interactions and microbial protective responses. In addition to dissolution and Zn^2+^‐mediated toxicity through particle–cell interactions, ZnO NPs can induce microbial stress through ROS generation, the latter being particularly relevant under illumination because of the photocatalytic properties of ZnO.

Comparable microbial responses have also been reported for other photoactive engineered nanoparticles, particularly TiO_2_ NPs. Long‐term TiO_2_ NP exposure has been shown to alter biological nutrient‐removal performance and restructure activated‐sludge bacterial communities, with effects depending on nanoparticle concentration and exposure duration (Zheng, Chen, et al. 2011; Li et al. 2014, 2017). TiO_2_ NP exposure can also affect microbial viability and stimulate extracellular protective responses, including enhanced EPS secretion and changes in biofilm thickness and density (Mathur et al. 2017). These studies demonstrate that nanoparticle‐induced oxidative and surface‐associated stress can produce substantial changes in microbial physiology and community organisation even for comparatively water‐stable nanomaterials such as TiO_2_.

Biofilms may respond differently from suspended microbial communities because the extracellular matrix acts as both a physical barrier and a reactive sink for nanoparticles and dissolved metals. Microscopic studies have demonstrated substantial adsorption of ZnO NPs onto biofilm matrices, while penetration and biological inhibition may remain concentrated in the outer biofilm layers. For example, Hou et al. (2016) reported that exposure to 50 mg L^−1^ ZnO NPs inhibited oxygen respiration primarily within the outer approximately 200 μm of aerobic wastewater biofilms; scanning electron microscopy confirmed particle association with the biofilm, whereas ZnO dissolution and the resulting Zn^2+^ exposure were identified as major contributors to inhibition. Other biofilm studies similarly observed substantial ZnO NP accumulation at biofilm surfaces and highlighted the protective role of EPS in restricting nanoparticle penetration. More recent work has also shown that microbial biofilms can respond to ZnO exposure through changes in extracellular matrix production, cellular damage and stress‐associated metabolic pathways. These observations emphasise that ZnO NP effects in MBRs cannot be interpreted solely from nominal nanoparticle concentration because particle attachment, dissolution, Zn^2+^ complexation and EPS‐mediated protection may occur simultaneously.

Despite numerous studies on the short‐term toxicity of ZnO NPs in biological treatment systems, considerably less information is available regarding long‐term microbial adaptation and resilience during continuous nanoparticle exposure. Most previous studies have focused primarily on reactor performance indicators or short exposure periods ranging from several hours to a few weeks, often under batch conditions or relatively low operational complexity (Ge et al. 2011; Hou et al. 2016). Only limited information is available regarding the evolution of microbial communities during prolonged ZnO NP exposure in continuously operated MBR systems, particularly under exposure periods exceeding 100 days. Furthermore, relatively few studies have simultaneously investigated microbial community restructuring together with changes in EPS and SMP characteristics, despite the close relationship between microbial stress responses and extracellular polymer production. The mechanisms governing microbial adaptation, community restructuring, changes in taxonomic and phylogenetic richness, and shifts in dominant functional populations under long‐term nanoparticle stress therefore remain poorly understood. In particular, relatively few long‐term MBR studies have simultaneously related microbial community restructuring and extracellular responses to the transformation of ZnO NPs and the potential contribution of dissolved Zn^2+^, despite increasing evidence that ZnO dissolution is a major determinant of biological toxicity.

In addition, reported environmental concentrations of ZnO nanoparticles are generally low, typically ranging from ng/L to low μg/L levels in wastewater effluents and receiving waters (Gottschalk et al. 2009; Keller et al. 2013). Nevertheless, localised accumulation, industrial discharges, accidental releases or sludge‐associated concentration effects may result in substantially higher exposure levels within biological treatment systems. Therefore, this study employed progressively increasing ZnO NP concentrations as a controlled long‐term stress scenario rather than a dose–response experiment. The objective was to investigate microbial adaptation, community restructuring and extracellular responses under increasing nanoparticle stress, rather than to determine toxicity thresholds at environmentally relevant concentrations. The novelty of this study lies in its long‐term evaluation of microbial community restructuring under progressive ZnO NP exposure in a continuously operated anoxic–aerobic MBR system. Unlike many previous studies that examined short‐term toxicity or bulk reactor performance, this work links 144‐day nanoparticle exposure with compartment‐specific microbial community shifts, α‐diversity responses, dominant functional genera and extracellular stress indicators including LB–EPS, TB–EPS, HPLC–SEC and FEEM profiles. This integrated approach provides a clearer understanding of how nanoparticle stress reshapes microbial ecology and extracellular protective responses in engineered biological treatment systems.

The objective of this study was therefore to investigate the long‐term effects of ZnO nanoparticles on microbial community dynamics in submerged anoxic–aerobic MBRs operated for 144 days under continuous ZnO NP exposure. Particular emphasis was placed on evaluating shifts in microbial diversity, community structure, dominant functional genera and extracellular polymer production under nanoparticle stress. Changes in LB–EPS, TB–EPS, SMP characteristics, molecular weight (MW) distribution and fluorescence properties were also examined to better understand microbial adaptation mechanisms and extracellular responses associated with prolonged ZnO NP exposure.

## 2 Materials and Methods

### 2.1 Setup and Operation of MBR Systems

**FIGURE 1:**
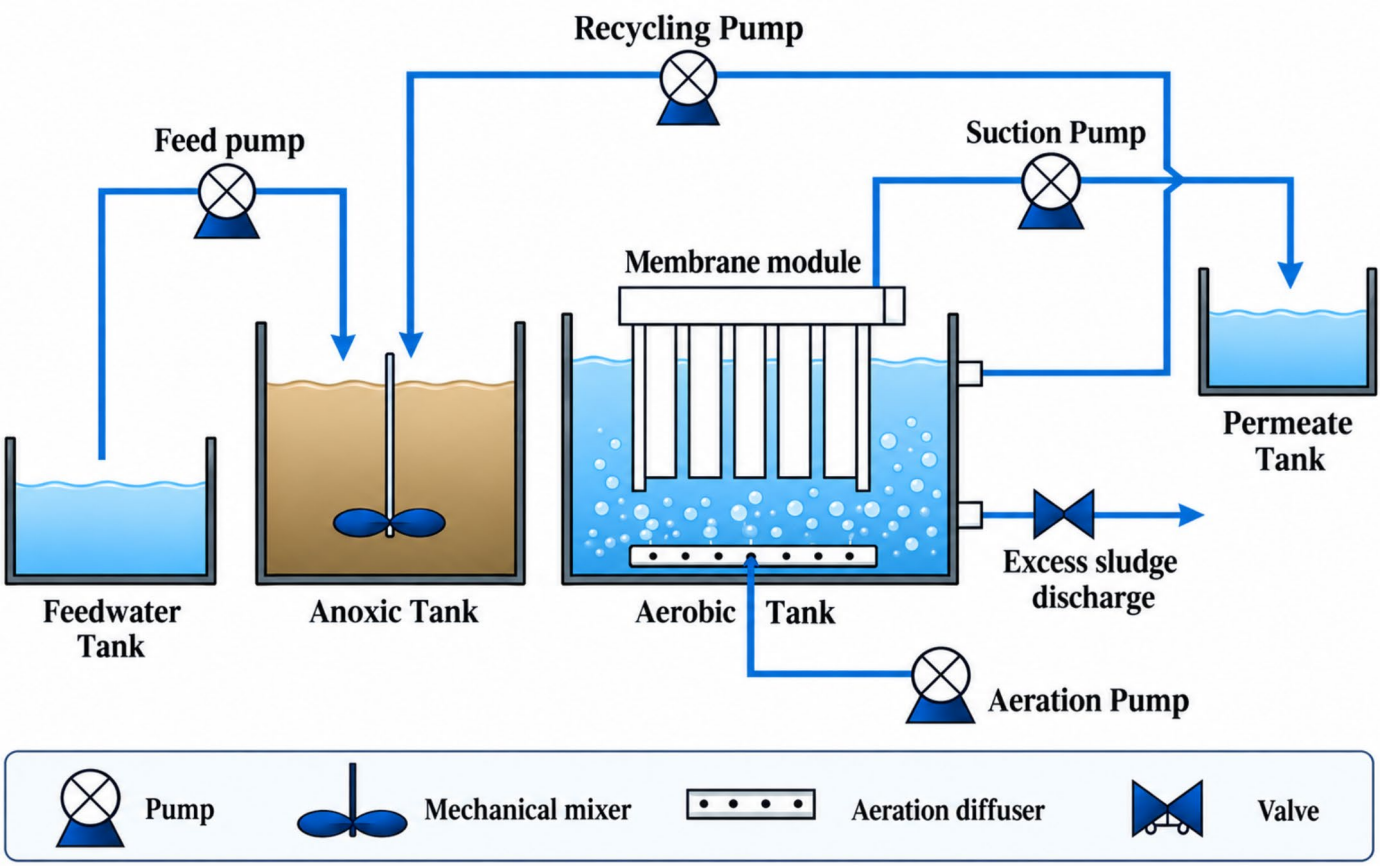
Schematic diagram of the MBR systems. *Note:* The system consists of a feedwater tank, anoxic tank, aerobic tank equipped with a membrane module, and a permeates tank. Mixed liquor is recycled from the aerobic tank to the anoxic tank. Permeate is collected by suction, and excess sludge is withdrawn from the aerobic tank.

Two parallel laboratory‐scale MBRs were operated under identical conditions, comprising a 3 L anoxic compartment connected to a 7 L aerobic compartment. A schematic representation of the experimental anoxic–aerobic MBR configuration is shown in Figure 1. A hollow fibre ultrafiltration membrane (ZeeWeed 500, GE Singapore) made of polyvinylidene fluoride with an effective membrane surface area of 565 cm^2^ and a nominal pore size of 0.04 μm was submerged in the aerobic tank. Activated sludge collected from the Ulu Pandan Wastewater Reclamation Plant (Singapore) was used as the inoculum source for both reactors.

The concentrations of the feed components in synthetic wastewater were as described by Zhang et al. (2017) (in mg/L): 356 sucrose (C_12_H_22_O_11_), 22 KH_2_PO_4_, 153 NH_4_Cl, 34 CaCl_2_, 640 NaHCO_3_, 30 CuSO_4_·5H_2_O, 20 MgSO_4_·7H_2_O, 20 FeSO_4_·7H_2_O, 58 CoCl_2_·6H_2_O, 150 H_3_BO_4_, 20 EDTA (C_10_H_16_N_2_O_8_) and 30 KI. The synthetic wastewater used in this study was designed to simulate typical municipal wastewater, containing a mixture of carbon (e.g., sucrose), nitrogen (e.g., NH_4_^+^–N), phosphorus (e.g., PO_4_^3−^–P) and essential trace elements. Synthetic wastewater was selected to maintain stable and reproducible operating conditions and to facilitate interpretation of nanoparticle‐induced changes in microbial activity and extracellular product formation. The MLSS and MLVSS concentrations were monitored (*n* = 3) throughout the 144‐day operation (Figure S2). Although gradual changes in biomass concentration occurred during long‐term operation, no abrupt biomass washout was observed, and both reactors remained within the typical operating range for laboratory‐scale MBRs. The hydraulic retention time (HRT) of 15 h is within the range commonly used for lab‐scale MBR studies and was selected to ensure stable biological treatment performance and sufficient contact time for nutrient removal processes. While shorter HRTs (e.g., 6–10 h) are often applied in full‐scale systems, longer HRTs are frequently used in experimental studies to: maintain stable microbial communities, reduce operational variability and allow clearer observation of stress responses (e.g., nanoparticle effects). Therefore, the selected HRT is not excessively long but represents a controlled operating condition suitable for mechanistic investigation, rather than direct replication of full‐scale operation.

The system was operated for 3 months to achieve steady state. Thereafter, the ZnO NPs were added into the influent wastewater to achieve a final concentration of 1.0 mg/L from Day 1, 10 mg/L from Day 49 and 50 mg/L from Day 96. The operation of two MBRs for the experiment lasted 144 days. The control reactor (MBR‐C) only received regular feed without any ZnO NPs during the entire experiment. This progressive exposure strategy was selected to investigate chronic microbial adaptation during continuous reactor operation and should not be interpreted as a conventional dose–response design. Consequently, the selected concentrations were intended to represent increasing stress conditions rather than environmentally representative exposure levels or toxicity thresholds. Both MBRs were operated under normal synthetic laboratory lighting throughout the 144‐day experimental period and were not maintained in darkness. No intentional UV irradiation or dedicated photocatalytic light source was applied during ZnO NP exposure.

### 2.2 Characterisation of ZnO Nanoparticles

The ZnO NPs (50%, w/w) were purchased from Sigma‐Aldrich (Singapore), and stock solutions (100 mg/L) were prepared. To minimise ZnO NP aggregation, ultrasonic treatment (30°C, 100 W, 40 kHz) for 30 min and shaking for 2 h were carried out to enhance their dispersion before the ZnO NPs suspension was used. The morphology of the ZnO NPs was examined using transmission electron microscopy (TEM) (JEOL JEM‐3010, Japan) (Figure S1), and particle size analysis was performed using a Zetasizer Nano Z90 (Malvern Instruments, UK); the mean particle size was 33 ± 8 nm (data not shown).

### 2.3 Determination of Protein and Polysaccharide Concentrations in EPS

SMPs and EPS were extracted following the method described by Mei et al. (2014) with minor modifications. Mixed liquor samples were first centrifuged at 6000*g* for 10 min to separate the biomass from the supernatant. The resulting supernatant was collected as the SMP fraction, representing the soluble organic compounds released by microorganisms into the mixed liquor.

The sludge pellet was then resuspended in 0.9% (w/v) NaCl solution, mixed by horizontal shaking and sonicated at 20 kHz for 2 min to extract loosely bound EPS (LB–EPS). Following sonication, the suspension was centrifuged at 8000*g* for 10 min, and the supernatant was collected as the LB–EPS fraction.

The remaining sludge pellet was again resuspended in 0.9% (w/v) NaCl solution, mixed by horizontal shaking, and heated at 60°C for 1 h to extract tightly bound EPS (TB–EPS). After thermal extraction, the suspension was centrifuged at 11,000*g* for 10 min, and the supernatant was collected as the TB–EPS fraction. The combined LB–EPS and TB–EPS fractions represented the total bound EPS. All collected supernatants (SMP, LB–EPS and TB–EPS) were filtered through 0.45 μm membrane filters before chemical analysis.

Protein and polysaccharide concentrations in the SMP, LB–EPS and TB–EPS fractions were determined using the modified Lowry method and the anthrone‐sulphuric acid method, respectively. Bovine serum albumin (BSA) and glucose were used as calibration standards for protein and polysaccharide quantification. To facilitate comparison among samples with different biomass concentrations, the concentrations of proteins and polysaccharides in the EPS fractions and the total SMP concentration were normalised to the mixed liquor volatile suspended solids (MLVSS) concentration and expressed as mg g^−1^ VSS.

### 2.4 SMP MW Distribution

A high‐performance size‐exclusion chromatograph (HP‐SEC) (1260 LC system, Agilent, Santa Clara, CA, USA) equipped with a PL Aquagel‐OH 8 lm MIXED‐M column was used to determine the overall MW distribution of organic compounds. Milli‐Q water was used as the mobile phase at a flow rate of 1 mL/min. Polyethylene glycols (PEGs) and polyethylene oxide standards with MW of 500 kDa, 70 kDa, 4 kDa, 600 Da and 106 Da were used to calibrate the column. MW was calculated based on a linear relationship between the log of MW (Da) and retention time (Rt) as shown in the below Equation (1):

(1) $$\log\;\left(\mathrm{MW}\right)=9.883-0.6748\;\left(\mathrm{Rt}\right)$$

### 2.5 Three‐Dimensional Fluorescence Excitation Emission Matrix (FEEM)

Three‐dimensional FEEM spectra were measured using a luminescence spectroscope (LS55, Perkin‐Elmer, Waltham, MA, USA). The spectrometer slits were set at 10 nm for both excitation and emission, and excitation wavelengths were increased from 220 to 600 nm in 10 nm steps; for each excitation wavelength, the emission was detected from 300 to 550 nm in 10 nm steps. The spectra were recorded at a scan rate of 10,000 nm min^−1^, using excitation and emission slit bandwidths of 10 nm, and the software FL Winlab Version 4.00.03 (Perkin Elmer) was used to handle the EEM data.

### 2.6 DNA Extraction and Polymerase Chain Reaction (PCR)

To investigate the impact of dosing 50 mg/L ZnO NPs on the microbial community, samples from the MLSS and membrane biofilm in both MBRs were collected for DNA extraction. The sludge biomass samples were centrifuged at 500 rpm for 10 min at 4°C, and the 500 mg pellet was collected. The tubular membrane slices were subsectioned into pieces of approximately 1 cm^2^ and subjected to DNA extraction by a modified bead‐beating method described previously (Oved et al. 2001). Genomic DNA from MLSS was extracted using a Fast DNA Spin Kit for Soil (MP Biomedicals, USA). The DNA concentrations were determined using an ND‐2000 spectrophotometer (Nanodrop Technologies Inc., Wilmington, DE, USA). Bacterial 16S ribosomal DNA was amplified in a DNA thermocycler (Eppendorf, Germany) using universal primers: 515F (5′‐GTGCCAGCMGCCGCGGTAA‐3′) and 806R (5′‐GGACTACVSGGGTATCTAAT‐3′), targeting the V4 hypervariable region of the 16S rRNA gene (Kozich et al. 2013). The PCR reaction system contained: 2 μL of template DNA, 10 μM each primer, 2.5 mM MgCl_2_, 200 μM dNTP, 5 μL of 10× PCR buffer and 0.5 U of Taq DNA polymerase (Fisher BioReagents, USA). PCR was performed with an initial denaturation at 94°C for 3 min and 20 cycles at 94°C for 1 min, followed by an annealing step at 55°C for 45 s, 72°C for 45 s and a final extension at 72°C for 10 min. The initial 16S rRNA gene PCR amplification was performed using 20 cycles in accordance with the Illumina MiSeq dual‐index amplicon sequencing protocol described by Kozich et al. (2013). This number of cycles was selected to minimise PCR‐induced amplification bias, chimaera formation and preferential amplification of dominant taxa while maintaining sufficient amplicon yield for high‐throughput sequencing. The sequencing generated 18,845–79,979 high‐quality reads per sample (average 45,010 reads), indicating that amplification was sufficient for reliable microbial community analysis (Kozich et al. 2013; Witzke et al. 2020).

### 2.7 Illumina Sequencing Analysis

The PCR products were sequenced at RTSF Genomics Core, Michigan State University. The samples were normalised using Invitrogen SequalPrep DNA normalisation plates; DNA recovered from the plates was pooled and given a final AmpureXP cleanup. Pooled amplicons were validated and quantified using Qubit dsDNA, Calliper LabChipGX DNA and Kapa Biosystems Illumina Library Quantification qPCR assays. The pool was loaded on a standard Illumina MiSeq flow cell (v2) and sequenced in a 2 × 250 bp paired‐end format with a 500 cycle v2 reagent cartridge. A PhiX DNA control library was added to the library mix to provide base composition diversity to the run. Base calling was done by Illumina real time analysis (RTA) v1.18.54, and the output of RTA was demultiplexed and converted to FastQ format using Bcl2fastq v1.8.4. The quantitative insights into microbial ecology (QIIME) pipeline (version 1.8.0) (Caporaso, Kuczynski, et al. 2010) was used for quality filtering, removal of chimeric sequences and downstream microbial community analyses using the default parameters. Low‐quality and chimeric reads were removed prior to downstream analysis. All samples were rarefied to 6730 sequences prior to alpha‐diversity analysis to standardise sequencing effort across samples. This depth allowed direct comparison of Faith's phylogenetic diversity (Faith's PD; PD_whole_tree in QIIME), Chao1 richness, and observed operational taxonomic units (OTUs) among samples. OTUs were picked using an open‐reference mode that combined closed‐reference and de novo, or open, OTU picking. OTUs undergoing closed‐reference picking were aligned with PyNAST (Caporaso, Bittinger, et al. 2010) against the May 2013 version of the GreenGenes database (DeSantis et al. 2006) at 97% similarity. Both alpha and beta diversity analyses were processed using QIIME.

### 2.8 Statistical Analysis

Differences in mixed liquor suspended solids (MLSS) and MLVSS between MBR‐C and MBR‐T were evaluated separately for each ZnO NP exposure period using paired two‐tailed Student's *t*‐tests, with measurements paired by sampling date. The analyses included 11 paired observations during the 1 mg L^−1^ exposure period (*n* = 11), 11 paired observations during the 10 mg L^−1^ exposure period (*n* = 11) and 14 paired observations during the 50 mg L^−1^ exposure period (*n* = 14). Quantitative EPS and SMP measurements were performed in triplicate (*n* = 3) and are presented as mean ± standard deviation (SD). Differences among the control, 1 mg L^−1^ ZnO NP and 50 mg L^−1^ ZnO NP conditions were evaluated using one‐way analysis of variance (ANOVA). Statistical significance was accepted at *p* < 0.05. Statistical analyses were performed using IBM SPSS Statistics version 20.

Differences in bacterial community composition among samples were evaluated using principal coordinate analysis (PCoA) based on Bray–Curtis dissimilarity calculated from genus‐level relative abundance data. To statistically assess differences in community composition between the control and ZnO NP‐treated reactors, a permutational multivariate ANOVA (PERMANOVA, *n* = 3) was performed using 999 permutations. The PERMANOVA pseudo‐*F* statistic was used to compare the variation among treatment groups relative to the variation within groups, while the coefficient of determination (*R* ^2^) was used to quantify the proportion of total community variation explained by the treatment. Statistical significance was determined from permutation‐derived *p*‐values, with differences considered significant at *p* < 0.05. Control and treatment *α*‐diversity values within each reactor compartment were compared using two‐sided Welch's *t*‐tests, with *p* < 0.05 considered statistically significant. For *α*‐diversity analysis, two biological replicate samples were available for each control and treatment condition within each reactor compartment (*n* = 2 per condition). Faith's PD (PD whole tree), Chao1 richness and observed OTUs were compared between control and treatment samples within each compartment using two‐sided Welch's *t*‐tests, which do not assume equal variances.

## 3 Results and Discussion

### 3.1 Reactor Performance

**FIGURE 2:**
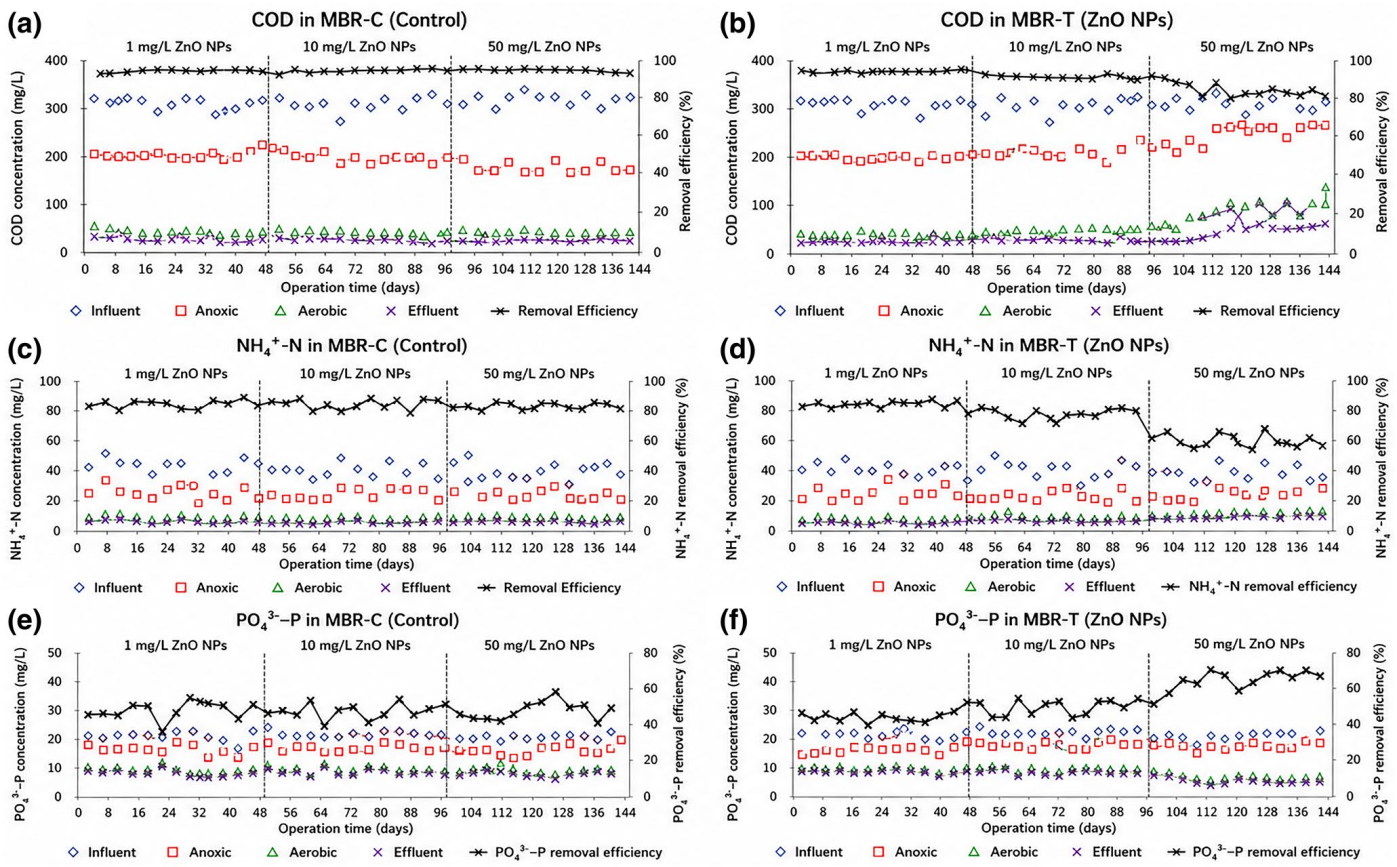
Pollutant removal performance in MBRs. (a) COD in MBR‐C; (b) COD in MBR‐T; (c) NH_4_^+^–N in MBR‐C; (d) NH_4_^+^–N in MBR‐T; (e) PO_4_^3−^–P in MBR‐C and (f) PO_4_^3−^–P in MBR‐T. ‘C’ represents control and ‘T’ represents treatment.

The effects of ZnO NP exposure on reactor performance are summarised in Figure 2. Pollutant removal performance gradually deteriorated as ZnO NP concentrations increased during long‐term operation. As shown in Figure 2a–b, the introduction of 1 mg/L ZnO NPs caused no significant change (*p* > 0.05) in COD removal compared with the control reactor, indicating that the microbial community remained relatively stable under low nanoparticle stress. However, prolonged exposure to higher ZnO NP concentrations led to a deterioration in treatment performance. At 10 mg/L ZnO NPs, COD removal decreased from 93.1% in the control reactor to 90.1%, indicating partial inhibition of microbial degradation activity.

More pronounced effects were observed for ammonium removal (Figure 2c,d). In the presence of 1 mg/L ZnO NPs, NH_4_^+^–N removal remained relatively stable at 85.0% ± 2.5%, suggesting that this concentration was within the tolerance range of the activated sludge microbial community. In contrast, exposure to 10 and 50 mg/L ZnO NPs significantly reduced ammonium removal efficiencies to 78.2% ± 5.8% and 65.2% ± 5.7%, respectively (*p* < 0.05).

ZnO NP exposure resulted in measurable accumulation of dissolved Zn^2+^ in the aerobic supernatant (Table S1). Mean dissolved Zn^2+^ concentrations increased from 0.13 mg L^−1^ during exposure to 1 mg L^−1^ ZnO NPs to 2.9 and 6.9 mg L^−1^ during exposure to 10 and 50 mg L^−1^, respectively. Despite this increase, overall Zn removal remained high (approximately 93%–94%), indicating substantial retention of Zn within the MBR. Biosorption onto activated sludge accounted for approximately 71.4%, 62.3% and 82.6% of Zn removal at the three exposure concentrations, respectively. The progressive increase in dissolved Zn^2+^ demonstrates that ZnO dissolution occurred during reactor operation and supports the interpretation that ionic Zn^2+^ contributed to the observed microbial stress and inhibition of nitrification. At the same time, the high overall Zn removal and substantial sludge accumulation indicate that adsorption and aggregation of ZnO‐associated Zn within the biomass were important removal pathways, consistent with previous studies (Kiser et al. 2010; Lombi et al. 2012; Kaegi et al. 2013; Ma et al. 2014; Tan et al. 2015). SEM observations (Figure S3) further revealed pronounced particulate aggregates associated with the biomass under ZnO NP exposure. Concurrently, Zn accumulation in the sludge increased from approximately 0.8–9.1 mg Zn g^−1^ MLSS at 1 mg L^−1^ to 17.5–112 mg Zn g^−1^ MLSS at 10 mg L^−1^ and 154–595 mg Zn g^−1^ MLSS at 50 mg L^−1^ (Table S1). SEM–EDX analysis further demonstrated substantial Zn enrichment of the exposed biomass, reaching 14.6 wt% Zn at the highest exposure level (Figure S4). These SEM–EDX observations, together with the high overall Zn removal (approximately 93%–94%) and substantial Zn accumulation in the sludge, provide experimental evidence for the retention of Zn‐containing particulate material by the activated sludge. Because ZnO NP aggregation, dissolution and speciation were not monitored continuously, the proportion of Zn remaining as intact nanoparticles during reactor operation cannot be determined. The observed biological responses therefore cannot be attributed exclusively to the nanoscale form of ZnO. Considering the progressive accumulation of Zn in the biomass, the measured dissolved Zn^2+^ concentrations and the known dissolution and aggregation behaviour of ZnO NPs in aqueous matrices, the effects observed in this study likely represent combined Zn‐associated stress, including dissolved Zn^2+^ toxicity and biomass‐associated Zn exposure. The stronger biological effects accompanying increasing Zn accumulation suggest that Zn burden was an important driver of toxicity; however, the simultaneous increase in exposure concentration and duration prevents these factors from being separated quantitatively. Accordingly, the present experiment should be interpreted as a long‐term progressive‐exposure study rather than an independent concentration–response assessment.

In addition to dissolution‐mediated Zn^2+^ toxicity and particle‐associated interactions, ROS generation may have contributed to the observed microbial stress because ZnO is photoactive. The reactors were operated under normal synthetic laboratory lighting without intentional UV irradiation; therefore, photocatalytic ROS production was not specifically induced or quantified in this study. Consequently, the relative contribution of light‐mediated ROS generation to the observed microbial responses cannot be separated from Zn^2+^‐ and particle‐associated effects.

Phosphorus removal also exhibited fluctuations under ZnO NP exposure (Figure 2e,f), suggesting that nanoparticle stress affected phosphorus‐accumulating microorganisms and nutrient transformation pathways. The MLSS concentrations in MBR‐C and MBR‐T were comparable during the initial 1 mg L^−1^ ZnO NP exposure period, averaging 2.849 ± 0.137 and 2.620 ± 0.299 g L^−1^, respectively, with no statistically significant difference between the reactors (paired two‐tailed *t*‐test, *p* = 0.064) (Figure S2 and Table S2). However, MLSS became significantly lower in MBR‐T during exposure to 10 mg L^−1^ ZnO NPs, with mean concentrations of 3.228 ± 0.145 g L^−1^ in MBR‐C and 2.515 ± 0.136 g L^−1^ in MBR‐T (*p* = 3.64 × 10^−6^). This difference became more pronounced during the 50 mg L^−1^ exposure period, when MLSS averaged 3.586 ± 0.249 g L^−1^ in MBR‐C compared with 2.054 ± 0.306 g L^−1^ in MBR‐T (*p* = 5.31 × 10^−8^). These results indicate that prolonged exposure to elevated ZnO NP concentrations was associated with a progressive reduction in suspended biomass in the treated reactor. Despite gradual decreases in MLSS and MLVSS in the ZnO NP‐dosed reactor during prolonged exposure (Figure S2), biomass concentrations remained within the normal operating range, and no biomass washout occurred.

### 3.2 EPS and SMP Production by Bacteria

Long‐term exposure to ZnO nanoparticles substantially affected the production and composition of EPS and SMPs in the MBR system (Table 1). Both LB–EPS and TB–EPS fractions increased progressively with increasing ZnO NP concentration, indicating that microorganisms responded to nanoparticle stress by enhancing extracellular polymer production. Compared with the control reactor, exposure to 1 and 50 mg/L ZnO NPs increased SMP concentrations from 6.20 ± 0.47 mg g^−1^ MLVSS to 11.22 ± 1.21 (*p* < 0.05) and 23.06 ± 1.97 mg g^−1^ MLVSS (*p* < 0.05), respectively (Table 1). Similar increases were observed for EPS‐associated proteins and polysaccharides, particularly in the TB–EPS fraction, where protein concentrations increased from 76.1 ± 5.3 to 105.3 ± 8.5 mg g^−1^ MLVSS and polysaccharides from 42.7 ± 3.8 to 54.3 ± 4.6 mg g^−1^ MLVSS under 50 mg/L ZnO NP exposure (*p* < 0.05).

**TABLE 1:** Effect of ZnO NPs on the production of loosely bound (LB) and tightly bond (TB) EPS and SMPs.

	LB–EPS (mg/g VSS)		TB–EPS (mg/g VSS)		SMP (mg/g VSS)
	Protein	Polysaccharide	Protein	Polysaccharide	
Control	27.8 ± 2.4	7.6 ± 1.1	76.1 ± 5.3	42.7 ± 3.8	6.20 ± 0.47
1 mg/L ZnO NPs	29.4 ± 3.9	8.8 ± 1.3	83.8 ± 6.8	45.5 ± 3.1	11.22 ± 1.21*
50 mg/L ZnO NPs	36.3 ± 4.7*	12.1 ± 1.8*	105.3 ± 8.5*	54.3 ± 4.6*	23.06 ± 1.97*

*Note:* Values are presented as mean ± SD of triplicate analyses.

* Significantly different from the control (*p* < 0.05).

Enhanced EPS secretion indicates that microorganisms responded to ZnO NP exposure by strengthening extracellular protective barriers. EPS matrices can reduce nanoparticle toxicity by binding metal ions, adsorbing contaminants and decreasing direct interaction between ZnO NPs and microbial cells (Sheng et al. 2010; Ma et al. 2014). This protective response is particularly relevant for ZnO because the EPS matrix can interact with both particulate ZnO and dissolution‐derived Zn^2+^. Biofilm studies have shown that ZnO NPs can accumulate on extracellular matrices while their penetration into deeper biofilm regions is restricted, and negatively charged functional groups within EPS can bind dissolved Zn species. Consequently, the increase in LB–EPS and particularly TB–EPS observed here may have represented a combined response to direct nanoparticle contact and increasing dissolved‐Zn exposure. Such sequestration may partly protect embedded cells, while simultaneously concentrating Zn‐containing material within sludge flocs and membrane‐associated biofilms. The observed enrichment of polysaccharides in both LB–EPS and TB–EPS fractions indicates that microorganisms preferentially increased the production of carbohydrate‐rich extracellular matrices under ZnO NP exposure. The increased polysaccharide fraction likely improved floc stability and formed diffusion‐limiting barriers that reduced nanoparticle penetration and Zn^2+^ exposure. Similar increases in EPS production under nanoparticle stress have previously been reported in activated sludge and MBR systems exposed to ZnO, TiO_2_ and Ag nanoparticles (Ye et al. 2021; Wang and Chen 2016). Mathur et al. (2017) found that TiO_2_ NP exposure enhanced EPS production and altered biofilm thickness and density, suggesting that increased extracellular polymer secretion represents a broader microbial protective response to nanoparticle stress. In both ZnO‐ and TiO_2_‐exposed systems, the EPS matrix may reduce direct particle–cell contact while modifying nanoparticle retention and microbial interactions within the biofilm. The stronger increase observed in TB–EPS compared with LB–EPS further suggests that prolonged nanoparticle exposure promoted structural adaptation of microbial aggregates and biofilms rather than only short‐term stress responses.

**FIGURE 3:**
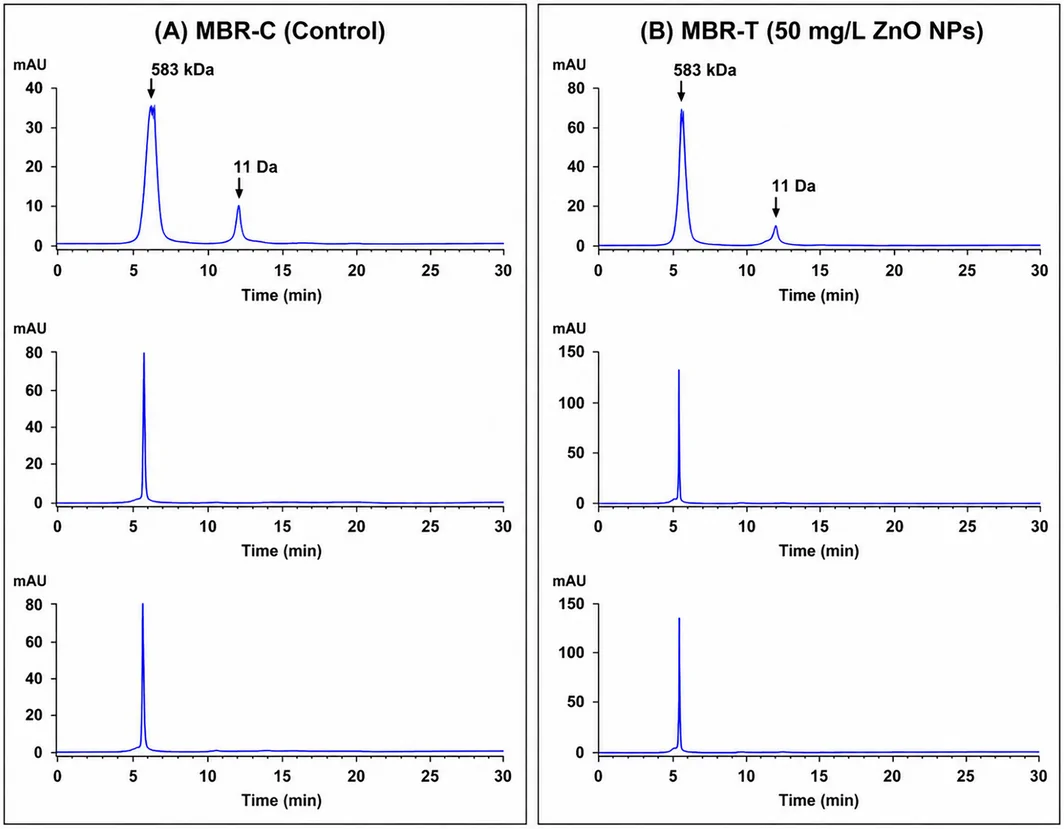
The molecular weight (MW) distribution of SMPs using high performance—size exclusion chromatograph (HP‐SEC): (A) MBR‐C without the addition of ZnO NPs and (B) MBR‐T under exposure to 50 mg/L ZnO NPs. ‘C’ represents control and ‘T’ represents treatment. Top: anoxic tank, middle: aerobic tank, bottom: effluent.

Changes in SMP MW distribution further supported the occurrence of stress‐induced extracellular transformations. HPLC–SEC analysis (Figure 3) revealed a clear increase in high‐molecular‐weight SMP fractions in the ZnO NP‐exposed reactor. In particular, the peak corresponding to approximately 583 kDa increased substantially in the anoxic compartment of MBR‐T compared with the control reactor, with absorbance intensities increasing from approximately 50 to 90 mAU. Similar trends were also observed in the aerobic compartment and effluent. The accumulation of high‐MW fractions suggests enhanced release of biopolymers and intracellular materials under nanoparticle stress. Previous studies have shown that toxic compounds and oxidative stress can promote cell lysis and release of proteinaceous and polymeric materials into the bulk liquid (Aquino and Stuckey 2004). High‐MW SMP fractions are often associated with proteins, polysaccharides and humic‐like macromolecules, which reflect microbial stress responses and extracellular organic matter accumulation in biological treatment systems (Jarusutthirak and Amy 2006).

**FIGURE 4:**
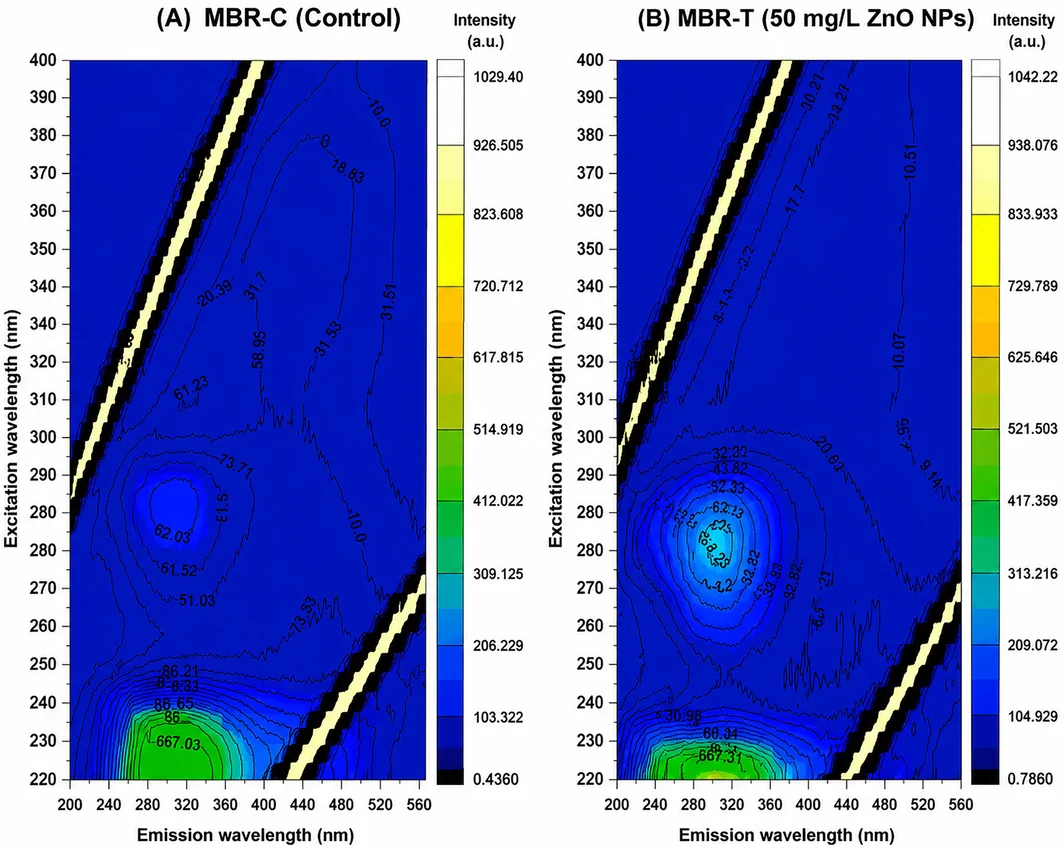
Three‐dimensional fluorescence excitation‐emission matrix (FEEM) contour maps of SMPs in MBR‐C and MBR‐T (A) in the aerobic MBR‐C and (B) in the aerobic MBR‐T at 50 mg/L ZnO NP concentration. ‘C’ represents control and ‘T’ represents treatment.

The FEEM spectra shown in Figure 4 also demonstrated substantial changes in SMP composition following ZnO NP exposure. Three major fluorescence regions were identified in both reactors. Region A (Ex/Em 225/360 nm) and Region B (Ex/Em 275/350 nm) corresponded mainly to tryptophan‐like and protein‐like substances, whereas Region C (Ex/Em 240/400 nm) was associated with fulvic acid‐like compounds (Wang et al. 2009). Compared with the control reactor, the ZnO NP‐exposed reactor exhibited markedly stronger fluorescence intensities in all three regions, particularly for protein‐like and fulvic acid‐like substances. These results indicate that ZnO NP exposure stimulated the accumulation of proteinaceous and humified SMP fractions.

The increase in protein‐like fluorescence may be attributed to enhanced microbial metabolism, secretion of stress‐related proteins and release of intracellular constituents following membrane damage and biomass decay. Similarly, the enrichment of fulvic acid‐like substances suggests increased transformation and humification of dissolved organic matter under prolonged nanoparticle exposure. Comparable increases in protein‐like and humic‐like SMP fractions have been reported under toxic or oxidative stress conditions in biological wastewater treatment systems (Baker 2001; Wang et al. 2009). Such compounds are generally more refractory and indicate increased accumulation of dissolved microbial products under prolonged nanoparticle stress.

Overall, the combined EPS, HPLC–SEC and FEEM analyses indicate that prolonged ZnO NP exposure induced substantial microbial stress responses and extracellular polymer production. The results suggest that microbial communities adapted to nanoparticle stress by increasing EPS synthesis and altering SMP composition, leading to the accumulation of high‐MW proteinaceous and fulvic‐like substances. These extracellular responses likely contributed to microbial protection and enhanced system resilience during prolonged nanoparticle exposure.

### 3.3 The Impact of ZnO NPs on the Microbial Community

After removing low‐quality sequences and chimaeras, 18,845–79,979 reads were obtained for each sample, with an average of 45,010 sequences. A total of 243–665 OTUs were identified per sample at a 97% similarity threshold, with 38 OTUs shared across all samples. Following quality filtering, sequencing depth varied among samples from 18,845 to 79,979 reads. To eliminate potential bias caused by unequal sequencing effort, all samples were subsequently rarefied to 6730 sequences prior to calculation of α‐diversity metrics (Table S3). Archaeal OTUs consisted mainly of methanogens, accounting for less than 1.5% of the total microbial community and averaging only 0.04% per sample. Therefore, archaea are not discussed further in this study.

**FIGURE 5:**
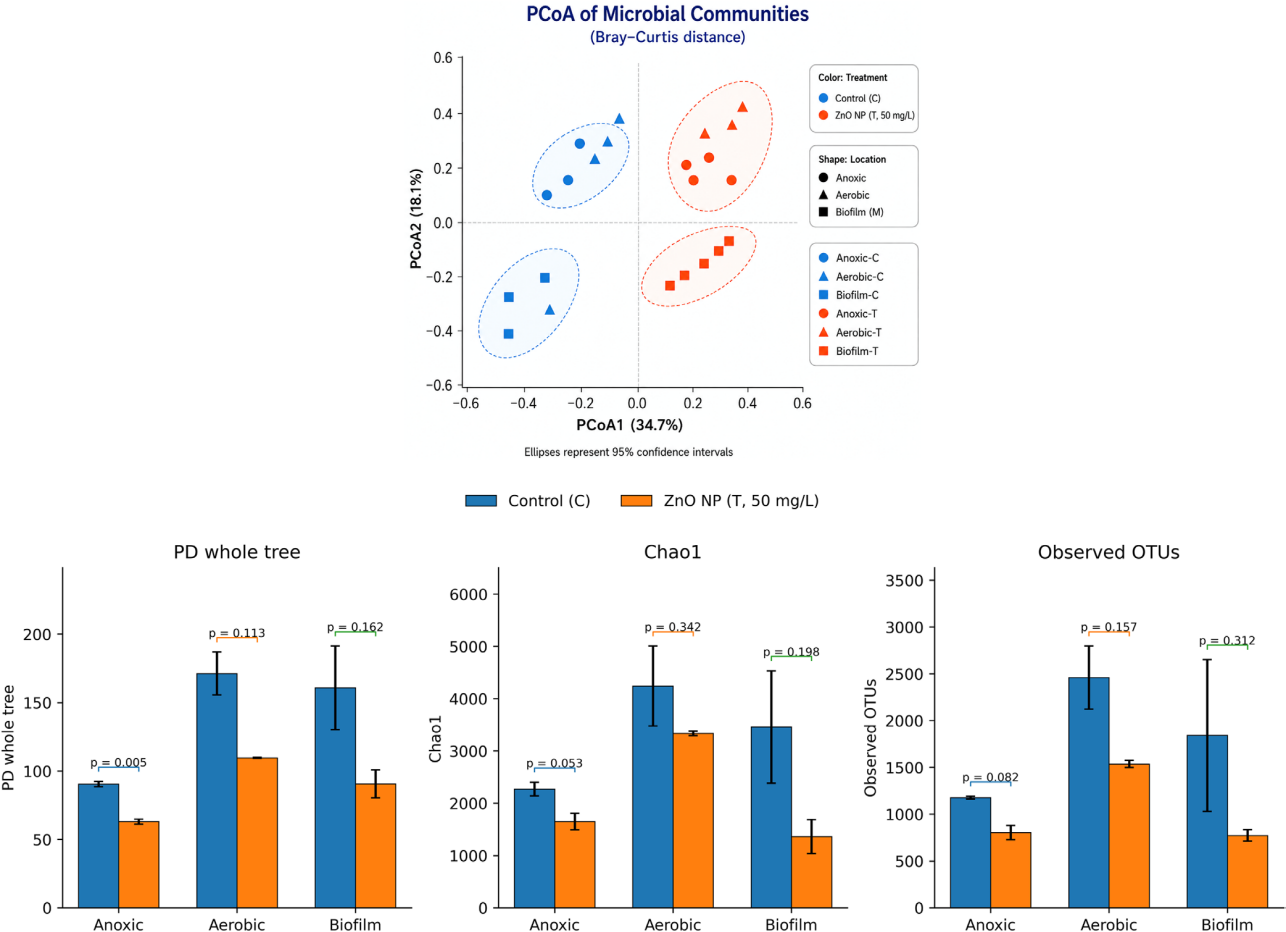
(Top) Microbial community response to ZnO NP exposure. (A) Principal coordinate analysis (PCoA) showing treatment‐related shifts in microbial community composition across anoxic, aerobic and biofilm compartments under ZnO NP exposure. Ellipses represent 95% confidence intervals. Differences in microbial community composition between the control and ZnO NP‐treated reactors were significant according to PERMANOVA based on Bray–Curtis dissimilarity (pseudo‐*F* = 3.46, *R* ^2^ = 0.257, *p* = 0.012; 999 permutations). (Bottom) α‐Diversity of microbial communities in the anoxic, aerobic and membrane biofilm compartments of the control (C) and ZnO NP‐exposed (T, 50 mg L^−1^) MBRs, assessed using Faith's phylogenetic diversity (PD whole tree), Chao1 richness and observed OTUs. Bars represent mean ± SD (*n* = 3). Statistical differences between control and treatment samples within each compartment were evaluated using two‐sided Welch's *t*‐test; *p* < 0.05 was considered statistically significant.

Distinct community compositions were observed across the anoxic, aerobic and biofilm compartments in the control reactor, indicating strong spatial differentiation within the MBR system. Under ZnO NP exposure, a clear restructuring of the microbial community occurred across all compartments. Such restructuring reflects the broad‐spectrum antimicrobial activity of ZnO nanoparticles, which affects entire microbial consortia rather than individual species (Dong et al. 2014). PCoA based on Bray–Curtis dissimilarity revealed a clear separation between the microbial communities of the control and ZnO NP‐treated reactors (Figure 5 top). This observation was supported by PERMANOVA, which confirmed that community composition differed significantly between treatments (pseudo‐*F* = 3.46, *R* ^2^ = 0.257, *p* = 0.012), indicating that long‐term ZnO NP exposure significantly altered the bacterial community structure. ZnO NP exposure was associated with lower phylogenetic and taxonomic richness across the three MBR compartments (Figure 5 Bottom). PD_whole_tree decreased from 90.42 ± 1.89 to 62.98 ± 1.81 in the anoxic compartment (*p* = 0.0045), representing a significant reduction in PD. Lower mean PD_whole_tree values were also observed in the aerobic and membrane‐biofilm communities, although these differences were not statistically significant (*p* > 0.05). Similarly, Chao1 richness and observed OTUs were consistently lower in the ZnO NP‐exposed communities than in their corresponding controls. Chao1 decreased from 2271 ± 130 to 1649 ± 157 in the anoxic compartment, from 4242 ± 764 to 3338 ± 43 in the aerobic compartment and from 3458 ± 1074 to 1362 ± 325 in the membrane biofilm. Observed OTUs showed a comparable pattern, decreasing by approximately 32%, 37% and 58% in the anoxic, aerobic and membrane biofilm communities, respectively. However, these differences did not reach statistical significance, likely reflecting the limited number of biological replicates (*n* = 2). Collectively, these results indicate restructuring of the microbial community under prolonged ZnO NP exposure, with evidence of reduced PD in the anoxic compartment and generally lower taxonomic richness across reactor compartments.

Taken together, the β‐ and α‐diversity results indicate that long‐term ZnO NP exposure was associated with both community compositional restructuring and a general reduction in taxonomic and phylogenetic richness, rather than increased microbial diversification. The loss of richness may reflect selective pressure against Zn‐sensitive populations while allowing more tolerant taxa to persist under prolonged nanoparticle exposure. This interpretation is consistent with the taxonomic shifts described below and suggests that adaptation of the MBR community did not necessarily involve maintenance of its original microbial diversity. The progressive increase in ZnO NP concentration from 1 to 10 and subsequently 50 mg L^−1^ should nevertheless be considered when interpreting these responses. Because exposure concentration increased with operational time, the observed microbial changes represent the combined effects of prolonged exposure and increasing ZnO NP stress rather than an independent concentration–response relationship. Consequently, this study provides insight into microbial ecological restructuring during chronic ZnO NP exposure but does not establish quantitative toxicity thresholds.

In line with these results, the community structure was altered as some genera lost their predominance. *Nitrospira* was the most abundant genus in the aerobic stage, and its abundance dropped by almost 80% after dosing. The observed inhibition of functional groups such as nitrifiers is consistent with previous studies reporting that ZnO nanoparticles negatively affect biological nitrogen removal processes (Dong et al. 2014). It was also found that some key enzymes, e.g., dehydrogenase, ammonia monooxygenase, nitrite reductase, nitrite oxidoreductase and nitrate reductase were negatively affected by long‐term exposure to ZnO NPs (Wang et al. 2016). In a previous study on the effect of ZnO NPs on *Nitrosomonas europaea* , an ammonia‐oxidising bacterium, these authors revealed that positively charged NPs attached to these negatively charged cells via adsorption or electrostatic attraction, which facilitates cytotoxicity. NPs further disturb membrane‐associated metabolic activities, specifically membrane permeability regulation, and such effect was concentration dependent (Wu et al. 2017). Inhibition might also be due to the release of zinc ions from ZnO NPs dissolution, and/or the increase in the production of ROS (Zheng, Wu, et al. 2011). ZnO nanoparticles underwent partial dissolution in the reactor environment, leading to measurable concentrations of soluble Zn^2+^, which contributed to the observed biological effects (Table S1). The dissolution profile revealed that with 1 and 10 mg/L NPs, the Zn^2+^ level in the aerobic supernatant was 0.13 and 2.9 mg/L, and Zn removal by bioadsorption was around 71.4% and 62.3%, respectively, while the overall Zn removal was 93%. This finding is in good agreement with Tan et al. (2015) who found that activated sludge adsorption/settling accounted for 65.9% of the total Zn contents in an MBR with 10 mg/L ZnO NPs. The results also showed that ZnO NPs tended to aggregate in the mixed liquor due to interactions with biomass and EPS, which can influence their bioavailability and toxicity. These findings support the interpretation that both particulate ZnO and released Zn^2+^ ions play a role in affecting microbial activity and SMP production.

The concurrent reduction in microbial richness and decline of key functional taxa indicates that long‐term ZnO NP exposure imposed substantial selective pressure on the microbial community. Lower Faith's PD (PD whole tree), Chao1 and observed OTU values in the ZnO NP‐exposed communities suggest a loss of taxonomic and PD, potentially reflecting the suppression of Zn‐sensitive populations. This ecological restructuring was accompanied by a decline in ammonium removal, indicating that the loss of microbial diversity included functionally important populations involved in nutrient transformation. These findings therefore suggest that prolonged ZnO NP stress can simultaneously reduce microbial community diversity and impair biological treatment performance. Similar community‐level responses have been reported during TiO_2_ NP exposure. Long‐term studies have demonstrated shifts in activated‐sludge bacterial composition together with alterations in nutrient‐removal performance, particularly among populations involved in nitrogen transformation (Zheng, Chen, et al. 2011; Li et al. 2014, 2017). These observations support the interpretation that prolonged engineered‐nanoparticle exposure can act as a selective pressure that restructures wastewater microbial communities and alters the abundance of functionally important populations.

**FIGURE 6:**
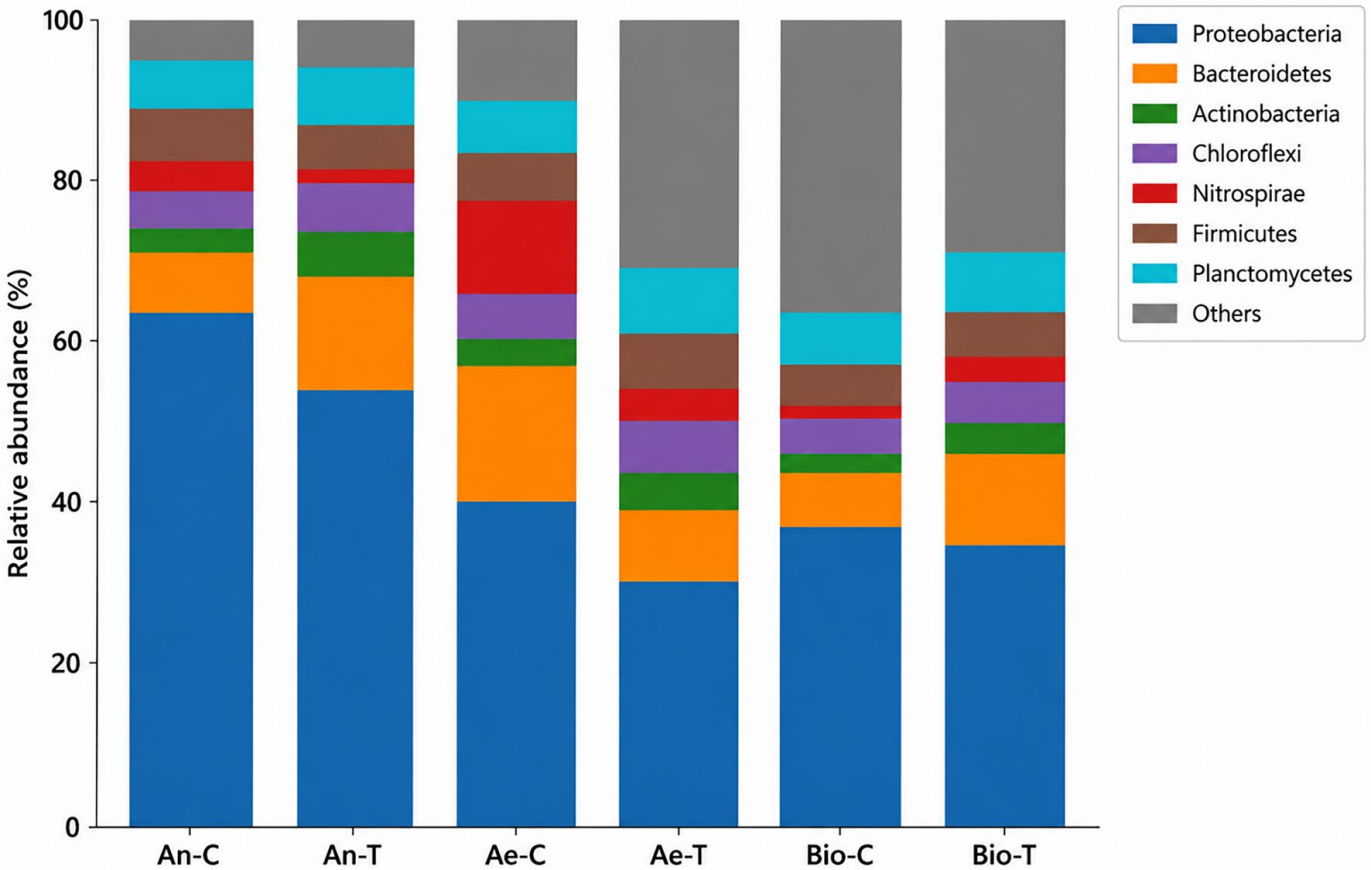
Changes in microbial community composition at the phylum level under ZnO nanoparticle exposure. Relative abundances of the dominant bacterial phyla in sludge samples from anoxic (An), aerobic (Ae) and membrane biofilm (Bio) compartments under control (C) and ZnO NP treatment (T, 10 mg/L) conditions. ZnO NP exposure resulted in a restructuring of the microbial community, characterised by a decrease in Proteobacteria and Nitrospirae and an increase in Bacteroidetes and other minor phyla. Minor taxa were grouped as ‘Others’ for clarity.

At the phylum level (Figure 6), Proteobacteria remained the dominant group in the control reactor, accounting for 65%, 40% and 37% in the anoxic, aerobic and biofilm compartments, respectively, followed by Bacteroidetes. Other phyla such as Planctomycetes, Actinobacteria, Nitrospirae, Chloroflexi, Bacteroidetes and Firmicutes were also present in appreciable abundances. Following ZnO NP exposure, a general decrease in Proteobacteria was observed (e.g., from 65% to 54% in the anoxic stage), along with reductions in Firmicutes and Chloroflexi. In contrast, several previously less dominant phyla, including Planctomycetes, Verrucomicrobia, Nitrospirae, Acidobacteria and Actinobacteria, increased in relative abundance, indicating selective enrichment of particular taxa within a substantially restricted community.

The decrease in Proteobacteria is particularly important because this phylum includes many organisms involved in carbon oxidation, denitrification and phosphorus cycling in activated sludge systems. Therefore, its reduction suggests a weakening of major functional groups responsible for nutrient transformation. In contrast, the enrichment of less dominant phyla such as Actinobacteria, Acidobacteria, Verrucomicrobia and Planctomycetes may reflect the selection of taxa with greater tolerance to oxidative stress, slower growth strategies or the ability to utilise complex organic substrates released during biomass decay and EPS/SMP transformation.

**FIGURE 7:**
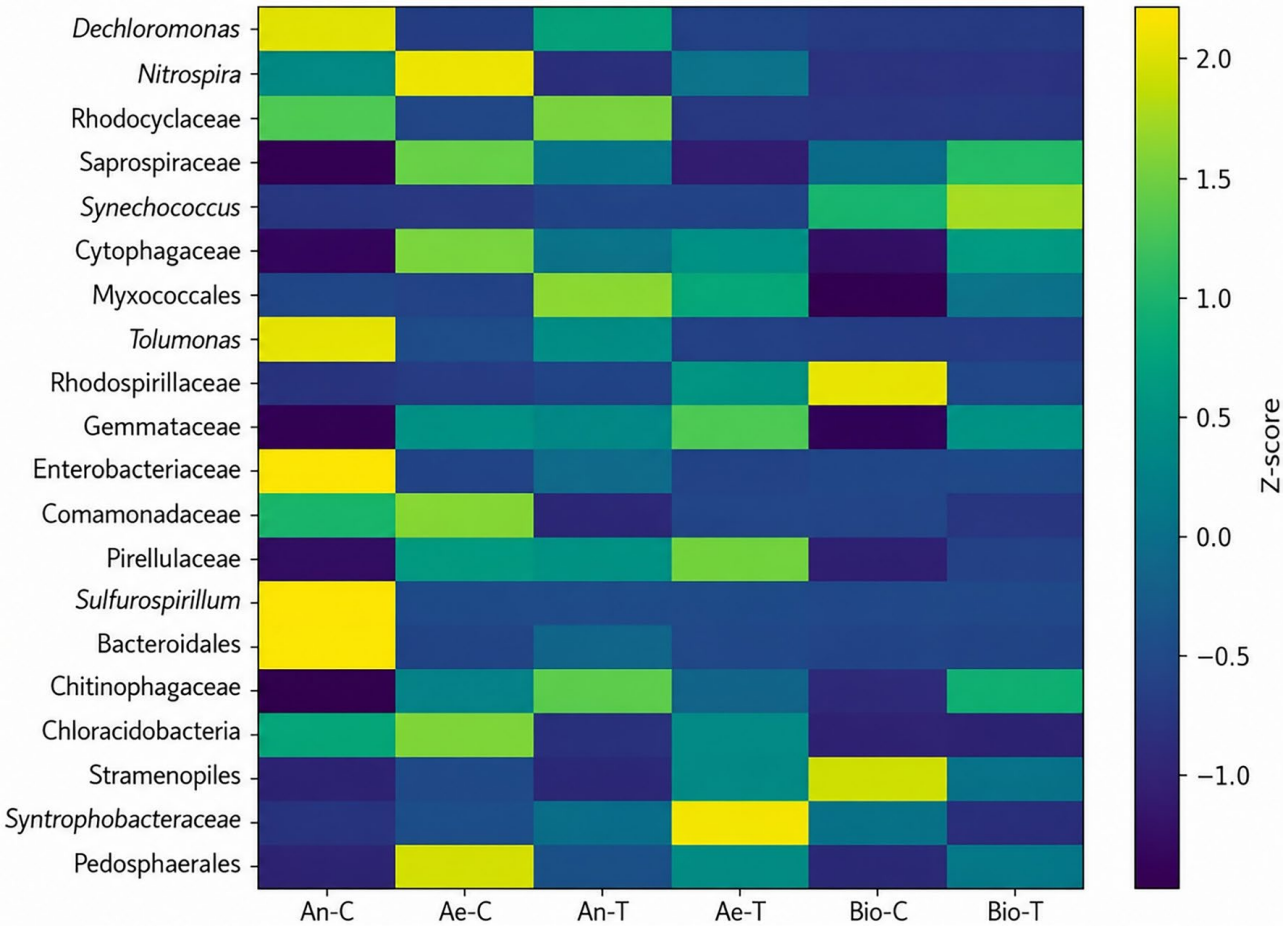
Heatmap of dominant bacterial taxa under ZnO nanoparticle exposure. Relative abundances of the dominant bacterial genera across anoxic (An), aerobic (Ae) and membrane biofilm (Bio) compartments under control (C) and ZnO NP treatment (T, 50 mg/L) conditions. Values represent mean abundances of replicate samples. Data are normalised using row‐wise *Z*‐scores to highlight relative differences across conditions. ZnO NP exposure induced substantial shifts in key functional genera, indicating restructuring of microbial communities under nanoparticle stress.

The genus‐level microbial community structure (Figure 7) revealed an overall restructuring of the microbial community under ZnO NP exposure across the anoxic, aerobic and biofilm compartments. Changes in the relative abundance of dominant genera indicate substantial restructuring of community composition. The genus‐level patterns shown in Figure 7 further highlight these community shifts. In the control reactor, *Dechloromonas* dominated the anoxic stage, while *Nitrospira* was the predominant genus in the aerobic compartment, reflecting typical functional stratification in MBR systems. After ZnO NP exposure, substantial changes in the relative abundance of these and other genera were observed, indicating a reorganisation of microbial community structure under nanoparticle stress. The contrasting responses of specific taxa between aerobic mixed liquor and biofilm communities highlight the distinct ecological niches within MBR systems. While suspended biomass is more directly exposed to nanoparticle‐induced stress, biofilms can provide protective microenvironments that mitigate toxicity and support the persistence or enrichment of sensitive taxa such as *Nitrospira*. Conversely, shifts in substrate availability and microbial interactions under ZnO NP exposure can differentially influence opportunistic groups such as Stramenopiles across these compartments.

The most dominant genus in the anoxic community was *Dechloromonas*, whose abundance decreased from 21% to 14%, while it increased from 0.8% to 5.4% in the aerobic community, implying a modest resilience when exposed to ZnO NPs. Members of *Dechloromonas* are facultative anaerobes with a broad spectrum of substrates as electron acceptors (Horn et al. 2005; Rizkallah et al. 2013; Wolterink et al. 2005). Recent studies also suggest that they are closely involved in Fe(II) oxidation (Chakraborty and Picardal 2013) and polyphosphate accumulation (Terashima et al. 2016). A relatively wide niche might have enabled *Dechloromonas* to survive with ZnO NPs in our MBR.

The responses of Rhodocyclaceae, Saprospiraceae and Cytophagaceae to ZnO NP exposure provide further insight into functional shifts within the microbial community. Members of the Rhodocyclaceae are well‐known denitrifying bacteria commonly enriched in anoxic zones of activated sludge systems, where they contribute to nitrogen removal through the utilisation of diverse organic substrates (Oehmen et al. 2010; Xia et al. 2018). The observed decrease in Rhodocyclaceae under ZnO NP exposure, particularly in the anoxic compartment, suggests that denitrifying populations are sensitive to nanoparticle‐induced stress, potentially due to oxidative damage or inhibition of key enzymes involved in nitrate and nitrite reduction. This decline is consistent with the overall reduction in functional nitrogen‐transforming populations under metal oxide nanoparticle exposure.

In contrast, Saprospiraceae exhibited enrichment, particularly in the biofilm compartment. Members of this family are typically associated with the degradation of complex organic matter, including proteins, polysaccharides, and other macromolecules, and several Saprospiraceae taxa are closely associated with activated‐sludge flocs (Kondrotaite et al. 2022, Xia et al. 2008). Their increased abundance under ZnO NP exposure likely reflects enhanced availability of biodegradable substrates resulting from cell lysis and SMP transformation. Furthermore, Saprospiraceae have been reported to tolerate fluctuating environmental conditions and may play a role in stabilising microbial communities under stress by participating in organic matter recycling.

Similarly, Cytophagaceae are known for their ability to degrade high‐molecular‐weight biopolymers such as polysaccharides and proteins, contributing to the breakdown and transformation of extracellular organic matter (Kirchman 2002). The moderate increase of Cytophagaceae in the biofilm under ZnO NP exposure suggests a shift towards communities capable of utilising stress‐derived substrates, including EPS and SMP components. Their enrichment may also be linked to increased polysaccharide production observed in EPS fractions, as these organisms are well adapted to environments rich in extracellular organic matrices.

Overall, the contrasting responses of these genera indicate that ZnO NP exposure suppresses key functional groups involved in nutrient removal (e.g., Rhodocyclaceae), while favouring heterotrophic populations involved in biomass degradation and organic matter transformation (e.g., Saprospiraceae and Cytophagaceae). This shift reflects a transition towards a more heterotroph‐dominated community structure under nanoparticle stress, with implications for both nutrient cycling and SMP dynamics in MBR systems.

The heatmap further indicates that ZnO NP exposure did not simply suppress the microbial community uniformly, but reorganised the relative dominance of taxa in a compartment‐specific manner. Genera associated with nutrient removal declined in suspended biomass, whereas taxa associated with polymer degradation, biomass turnover or stress tolerance were relatively enriched, particularly in the biofilm. This suggests a functional transition from a community dominated by nutrient‐removal specialists towards a more stress‐adapted and heterotrophic community.

**FIGURE 8:**
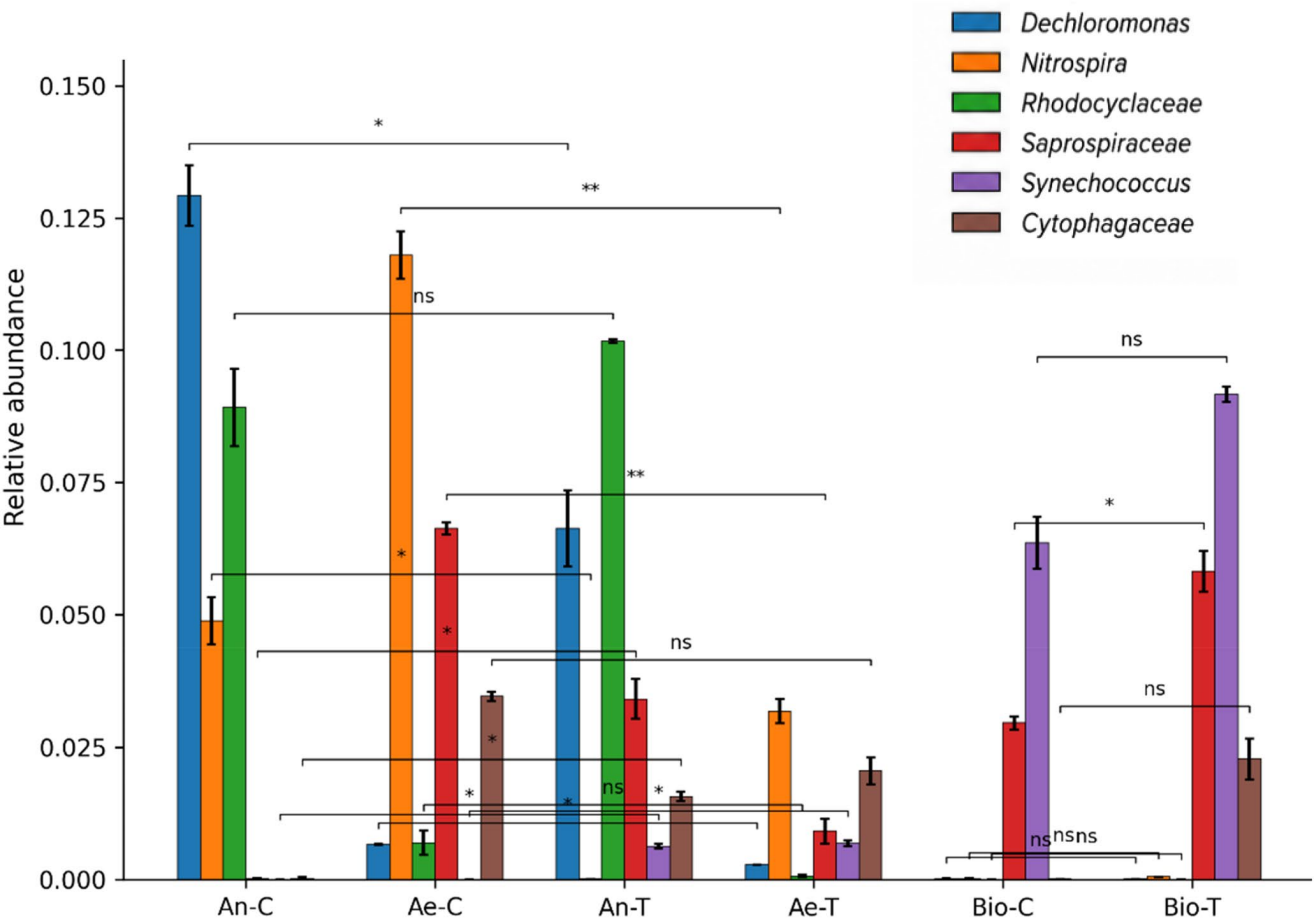
Relative abundance of the six most predominant bacterial taxa in control and ZnO NP‐dosed MBR systems. Relative abundances of the six dominant bacterial genera across anoxic (An), aerobic (Ae) and membrane biofilm (Bio) compartments under control (C) and ZnO NP treatment (T, 50 mg/L) conditions. Values represent mean abundances of replicate samples. * means *p* < 0.05, ** means *p* < 0.01, ns = not significant.

To better understand the functional implications of these changes, the responses of key genera are presented in Figure 8. Several nitrogen‐removal‐related genera were negatively affected by ZnO NP exposure. For example, the relative abundance of *Dechloromonas*, a well‐known denitrifying bacterium, decreased from 13% to 6.6%. Similarly, *Nitrospira*, a key nitrite‐oxidising bacterium, showed a marked decrease in the aerobic compartment (from 11% to 3%), consistent with the sensitivity of nitrifiers to ZnO NP‐induced stress and supporting previous findings of inhibited ammonia removal under nanoparticle exposure (Zhang et al. 2017). This is consistent with previous findings that ZnO nanoparticles can suppress nitrifying bacteria and reduce treatment efficiency (Wang et al. 2015; Ye et al. 2021). In contrast to the decline of some Rhodocyclaceae‐related taxa, PO_4_^3−^–P removal increased under 50 mg/L ZnO NP exposure (Figure 2). This suggests that phosphorus removal was not controlled solely by classical biological phosphorus‐accumulating organisms. The improvement may have resulted from abiotic phosphate removal mechanisms, including adsorption onto ZnO particles, complexation or precipitation with released Zn^2+^, and enhanced phosphate retention within EPS‐rich sludge flocs and membrane biofilms.

The relative abundances of the remaining dominant genera exhibited clear stage‐dependent variations under ZnO NP exposure. In the anoxic compartment, members of the *Rhodocyclaceae* decreased in abundance following ZnO NP addition, while *Saprospiraceae* and *Cytophagaceae* remained at relatively low levels with only minor changes. In contrast, the aerobic compartment showed a general decline in most genera under ZnO NP exposure, indicating a stronger inhibitory effect in suspended aerobic biomass.

In the biofilm compartment, a different trend was observed, with several genera showing increased abundance under ZnO NP exposure. Notably, *Synechococcus* and *Saprospiraceae* were enriched in the biofilm of the treated reactor, while *Cytophagaceae* also showed a moderate increase. These results suggest that biofilm‐associated communities are more resilient to ZnO NP stress, likely due to protective microenvironments, whereas suspended communities in the anoxic and aerobic stages are more sensitive to nanoparticle exposure.

The contrasting responses of certain taxa between aerobic mixed liquor and biofilm communities further highlight the distinct ecological niches within MBR systems. While suspended biomass is more directly exposed to nanoparticle‐induced stress, biofilms can provide protective microenvironments through EPS and diffusion limitations, which reduce effective nanoparticle exposure. This may explain the persistence or enrichment of sensitive taxa such as *Nitrospira* in biofilms despite their decline in suspended biomass. Conversely, changes in substrate availability and microbial interactions under ZnO NP exposure may differentially influence opportunistic groups such as *Synechococcus* and *Saprospiraceae* spp. across compartments.

Figure 8 further supports the conclusion that functional sensitivity differed among microbial guilds. Nitrifying and denitrifying organisms showed stronger inhibition than several heterotrophic taxa, suggesting that autotrophic and nutrient‐removal populations were more vulnerable to ZnO NP stress. This pattern is consistent with the observed decline in ammonium removal and indicates that long‐term ZnO NP exposure was associated with both loss of functionally important taxa and an overall reduction in taxonomic and phylogenetic richness.

Taken together, Figures 5, 6, 7, 8 show a consistent ecological pattern in which ZnO NP exposure substantially restructured microbial community composition while generally reducing taxonomic and phylogenetic richness. This restructuring was accompanied by a decline in several key functional populations involved in nitrogen transformation and by relative enrichment of selected heterotrophic and stress‐tolerant taxa, particularly within the biofilm. The microbial response should therefore be interpreted as selective ecological restructuring under prolonged Zn‐associated stress rather than as increased diversity, community convergence or ecological recovery. The concurrent decline in ammonium removal further indicates that these structural changes were accompanied by deterioration of important biological treatment functions.

## 4 Conclusions

This study demonstrated that long‐term exposure to ZnO nanoparticles significantly altered microbial community structure, extracellular polymer production and SMP characteristics in submerged anoxic–aerobic MBRs. Progressive ZnO NP exposure over 144 days resulted in substantial restructuring of bacterial communities across the anoxic, aerobic and biofilm compartments, accompanied by reduced nutrient removal performance, particularly for ammonium oxidation.

ZnO NP exposure increased the production of both LB–EPS and TB–EPS, with particularly strong increases in protein and polysaccharide concentrations within the tightly bound fraction. HPLC–SEC and FEEM analyses further revealed the accumulation of high‐molecular‐weight, protein‐like and fulvic‐like SMP fractions under nanoparticle stress, indicating enhanced extracellular polymer production, biomass turnover and transformation of dissolved organic matter. These extracellular responses likely functioned as protective mechanisms that mitigated nanoparticle toxicity through adsorption, diffusion limitation and stabilisation of microbial aggregates and biofilms.

Microbial community analysis showed that ZnO NP exposure significantly altered overall community composition and was associated with generally lower taxonomic and phylogenetic richness across reactor compartments. Faith's PD decreased significantly in the anoxic community, while Chao1 richness and observed OTUs showed lower mean values in ZnO NP‐exposed communities, although most of these differences were not statistically significant. These community‐level changes were accompanied by declines in key functional groups involved in nitrogen removal, whereas phosphorus removal appeared to involve additional abiotic and EPS‐mediated retention mechanisms. In contrast, heterotrophic and polymer‐degrading populations such as *Saprospiraceae* and *Cytophagaceae* became relatively enriched, particularly within biofilm communities. These results suggest that ZnO NP exposure shifted the microbial ecology from nutrient‐removal specialists towards a more stress‐adapted and heterotrophic community structure.

Overall, long‐term exposure to a progressively increasing Zn burden introduced as ZnO NPs resulted in substantial Zn accumulation within the MBR biomass and was associated with enhanced extracellular stress responses, microbial community restructuring, loss of taxonomic and phylogenetic richness, and deterioration of nutrient‐removal performance. Because ZnO NP speciation was not monitored throughout the experiment, the relative contributions of intact nanoparticles, dissolved Zn^2+^ and transformed or biomass‐associated Zn species could not be resolved. The observed effects should therefore be interpreted primarily as responses to Zn‐associated stress arising from ZnO NP exposure rather than as unequivocal evidence of nanoparticle‐specific toxicity. Future studies should investigate environmentally relevant nanoparticle concentrations, integrate metagenomic or metatranscriptomic approaches to better resolve functional responses, and evaluate the relationship between nanoparticle‐induced microbial restructuring, membrane fouling development, and treatment performance under full‐scale operating conditions.

## Author Contributions

**Dongqing Zhang:** conceptualization, investigation, writing – original draft, visualization, methodology, formal analysis, data curation. **Tao Yu:** validation, visualization, methodology, software, formal analysis, data curation, writing – original draft. **Antoine Prandota Trzcinski:** supervision, resources, project administration, formal analysis, writing – review and editing.

## Conflicts of Interest

The authors declare no conflicts of interest.

## Supporting information


**Figure S1:** Transmission electron microscopy (TEM) images of ZnO NPs in solution.
**Figure S2:** Temporal evolution of mixed liquor suspended solids (MLSS) and mixed liquor volatile suspended solids (MLVSS) in the control reactor (MBR‐C) and ZnO nanoparticle‐dosed reactor (MBR‐T) during the 144‐day experimental period.
**Figure S3:** Scanning electron microscopy (SEM) images of biomass from the control MBR (MBR‐C) and ZnO NP‐exposed MBR (MBR‐T).
**Figure S4:** Energy‐dispersive X‐ray spectroscopy (EDX) spectra of activated sludge collected from the control MBR (MBR‐C; left) and the ZnO NP‐exposed MBR (MBR‐T; right) at 50 mg L⁻^1^ ZnO NP exposure.
**Table S1:** Summary of dissolved Zn^2^⁺ concentrations, Zn removal by biosorption, overall Zn removal efficiency, and Zn accumulation in activated sludge during progressive exposure to 1, 10 and 50 mg L⁻^1^ ZnO NPs.
**Table S2:** Mean ± standard deviation (SD) (*n* = 3) of mixed liquor suspended solids (MLSS) and mixed liquor volatile suspended solids (MLVSS) in the control reactor (MBR‐C) and ZnO nanoparticle‐dosed reactor (MBR‐T) during the three operational stages of the experiment.
**Table S3:** Rarefaction depth and α‐diversity metrics of bacterial communities in the control and ZnO NP‐exposed MBRs.

## Data Availability

The data that support the findings of this study are available on request from the corresponding author. The data are not publicly available due to privacy or ethical restrictions.
