# Supplementary material for: Microbial Community Restructuring Under Zinc Oxide Nanoparticle Stress in Membrane Bioreactors
Source: Environ Microbiol Rep. 2026 Sep 16;18(5):e70413. doi: 10.1111/1758-2229.70413 (PMC13582292; doi:10.1111/1758-2229.70413)
Supplement: Supplementary file 1 [file EMI4-18-e70413-s001.docx]

|  |  |  |
| --- | --- | --- |

**Figure S1**. Transmission electron microscopy (TEM) images of ZnO NPs in solution. The particle sizes ranged from 15-47 nm, with a mean size of 33± 8 nm (n=107)

| a) | b) |
| --- | --- |
| c) | d) |

B

A

C

**Figure S2.** Temporal evolution of mixed liquor suspended solids (MLSS) and mixed liquor volatile suspended solids (MLVSS) in the control reactor (MBR-C) and ZnO nanoparticle-dosed reactor (MBR-T) during the 144-day experimental period.

**Figure S3.** Scanning electron microscopy (SEM) images of biomass from the control MBR (MBR-C) and ZnO NP-exposed MBR (MBR-T). (A, C) Control biomass from MBR-C; (B) biomass exposed to 10 mg L⁻¹ ZnO NPs; and (D) biomass exposed to 50 mg L⁻¹ ZnO NPs. SEM images were acquired at ×20,000 magnification with a 1 µm scale bar. ZnO NP-exposed biomass exhibited pronounced particulate aggregates associated with the biomass surface compared with the relatively smooth morphology observed in the control.

b)

a)

**Figure S4.** Energy-dispersive X-ray spectroscopy (EDX) spectra of activated sludge collected from the control MBR (MBR-C; left) and the ZnO NP-exposed MBR (MBR-T; right) at 50 mg L⁻¹ ZnO NP exposure. The MBR-T sludge exhibited a pronounced Zn signal, corresponding to 14.6 wt% Zn, confirming substantial Zn accumulation within the exposed biomass.

d)

c)

**Table S1.** Summary of dissolved Zn²⁺ concentrations, Zn removal by biosorption, overall Zn removal efficiency, and Zn accumulation in activated sludge during progressive exposure to 1, 10, and 50 mg L⁻¹ ZnO NPs.

^a^ Calculated as (Influent – Supernatant)/Influent x 100%

^b^ Calculated as (Influent – Effluent)/Influent x 100%

^c^ Zn accumulation in sludge normalized to mixed liquor suspended solids (MLSS); initial value measured before exposure (day 0). Values represent average ± standard deviation of triplicate measurements.

| **ZnO NP concentration (mg/L)** | **Mean dissolved Zn²⁺ in aerobic supernatant (mg/L)** | **Zn removal by biosorption^a^ (%)** | **Overall Zn removal efficiency^b^ (%)** | **Zn accumulation in sludge (mg/g-MLSS)^c^** | |
| --- | --- | --- | --- | --- | --- |
|  |  |  |  | **Initial** | **Final** |
| 1 | 0.13 ± 0.03 | 71.4 | 93 ± 2 | 0.8 | 9.1 |
| 10 | 2.9 ± 0.6 | 62.3 | 93 ± 2 | 0.8 | 17.5–112 |
| 50 | 6.9 ± 1.5 | 82.6 | 94 ± 2 | 0.8 | 154–595 |

**Table S2.** Mean ± standard deviation (SD) (n=3) of mixed liquor suspended solids (MLSS) and mixed liquor volatile suspended solids (MLVSS) in the control reactor (MBR-C) and ZnO nanoparticle-dosed reactor (MBR-T) during the three operational stages of the experiment. * indicates a statistically significant difference between reactors at *p* < 0.05.

| ZnO NP exposure period | MLSS (Control) | MLSS (Treatment) | *p*-value | MLVSS (Control) | MLVSS (Treatment) | *p*-value |
| --- | --- | --- | --- | --- | --- | --- |
| 1 mg/L (Day 1–48) | 2.849 ± 0.137 | 2.620 ± 0.299 | 0.064 | 2.627 ± 0.153 | 2.566 ± 0.099 | 0.220 |
| 10 mg/L (Day 49–95) | 3.228 ± 0.145^*^ | 2.515 ± 0.136^*^ | 3.64 × 10^-6^ | 3.035 ± 0.182^*^ | 2.355 ± 0.098^*^ | 1.73 × 10^-6^ |
| 50 mg/L (Day 96–144) | 3.586 ± 0.249^*^ | 2.054 ± 0.306^*^ | 5.31 × 10^-8^ | 3.339 ± 0.179^*^ | 1.874 ± 0.224^*^ | 2.45 × 10^-9^ |

**Table S3.** Rarefaction depth and α-diversity metrics of bacterial communities in the control and ZnO NP-exposed MBRs.

|  | **Rarefied sequences** | **PD_whole_tree Ave.** | **chao1 Ave.** | **observed_otus Ave.** |
| --- | --- | --- | --- | --- |
| Anoxic-C1 | 6730 | 89.082 | 2178.914 | 1165.7 |
| Anoxic-C2 | 6730 | 91.753 | 2363.412 | 1187 |
| Anoxic-T1 | 6730 | 61.696 | 1537.931 | 750 |
| Anoxic-T2 | 6730 | 64.255 | 1759.345 | 857.8 |
| Aerobic-C1 | 6730 | 182.338 | 4782.754 | 2697.4 |
| Aerobic-C2 | 6730 | 160.179 | 3702.186 | 2219.5 |
| Aerobic-T1 | 6730 | 109.884 | 3367.903 | 1509.1 |
| Aerobic-T2 | 6730 | 109.436 | 3307.354 | 1564 |
| Membrane-C1 | 6730 | 182.375 | 4217.466 | 2415 |
| Membrane-C2 | 6730 | 139.175 | 2698.233 | 1268.3 |
| Membrane-T1 | 6730 | 83.363 | 1132.72 | 729.1 |
| Membrane-T2 | 6730 | 97.765 | 1591.845 | 816.1 |
